# Improving clinical and epidemiological predictors of Buruli ulcer

**DOI:** 10.1371/journal.pntd.0006713

**Published:** 2018-08-06

**Authors:** Gilbert Adjimon Ayelo, Ghislain Emmanuel Sopoh, Jean-Gabin Houezo, René Fiodessihoue, Dissou Affolabi, Ange Dodji Dossou, Yves Thierry Barogui, Akpeedje Anita Carolle Wadagni, Didier Codjo Agossadou, Epco Hasker, Françoise Portaels, Bouke C. de Jong, Miriam Eddyani

**Affiliations:** 1 Centre de Dépistage et de Traitement de l’Ulcère de Buruli d’Allada, Ministry of Health, Allada, Benin; 2 Institut Régional de Santé Publique, University of Abomey Calavi, Ouidah, Benin; 3 Laboratoire de Référence des Mycobactéries, Ministry of Health, Cotonou, Benin; 4 Centre de Dépistage et de Traitement de l’Ulcère de Buruli de Lalo, Ministry of Health, Lalo, Benin; 5 Programme National de Lutte contre la Lèpre et l’Ulcère de Buruli, Ministry of Health, Cotonou, Benin; 6 Department of Public Health, Institute of Tropical Medicine, Antwerp, Belgium; 7 Department of Biomedical Sciences, Institute of Tropical Medicine, Antwerp, Belgium; University of Tennessee, UNITED STATES

## Abstract

**Background:**

Buruli ulcer (BU) is a chronic necrotizing infectious skin disease caused by *Mycobacterium ulcerans*. The treatment with BU-specific antibiotics is initiated after clinical suspicion based on the WHO clinical and epidemiological criteria. This study aimed to estimate the predictive values of these criteria and how they could be improved.

**Methodology/Principal findings:**

A total of 224 consecutive patients presenting with skin and soft tissue lesions that could be compatible with BU, including those recognized as unlikely BU by experienced clinicians, were recruited in two BU treatment centers in southern Benin between March 2012 and March 2015. For each participant, the WHO and four additional epidemiological and clinical diagnostic criteria were recorded. For microbiological confirmation, direct smear examination and IS*2404* PCR were performed. We fitted a logistic regression model with PCR positivity for BU confirmation as outcome variable. On univariate analysis, most of the clinical and epidemiological WHO criteria were associated with a positive PCR result. However, lesions on the lower limbs and WHO category 3 lesions were rather associated with a negative PCR result (respectively OR: 0.4, 95%CI: 0.3–0.8; OR: 0.5, 95%IC: 0.3–0.9). Among the additional characteristics studied, the characteristic smell of BU was strongest associated with a positive PCR result (OR = 16.4; 95%CI = 7.5–35.6).

**Conclusion/Significance:**

The WHO diagnostic criteria could be improved upon by differentiating between lesions on the upper and lower limbs and by including lesion size and the characteristic smell recognized by experienced clinicians.

## Author summary

Buruli ulcer (BU) is a neglected necrotizing skin disease caused by *Mycobacterium ulcerans*. The treatment with BU-specific antibiotics is initiated after clinical suspicion based on WHO diagnostic criteria. In this study we evaluated the WHO diagnostic guidelines for BU and how these criteria could be improved. A total of 224 patients presenting with skin lesions were recruited in two BU treatment centers in southern Benin between March 2012 and March 2015. Most of the clinical and epidemiological WHO criteria were associated with a confirmed BU diagnosis although lesions on the lower limbs were rather associated with a negative PCR result. Among the additional characteristics studied, the characteristic smell of BU was most strongly associated with a positive PCR result. The WHO diagnostic criteria could therefore be improved upon by discriminating between lesions on the upper and lower limbs and by including lesion size and the characteristic smell recognized by experienced clinicians. The volatiles responsible for this smell could serve as a Point-of-Care diagnostic test, useful for non-invasive confirmation during active case-finding activities, and for training of clinicians.

## Introduction

Buruli ulcer (BU) is a chronic necrotizing infectious disease of the skin caused by *Mycobacterium ulcerans*. After tuberculosis and leprosy, BU is the third most common mycobacterial disease worldwide [1,2]. BU has been reported in over 30 countries, typically in warm and humid intertropical regions where it predominantly affects children among poor and rural populations with difficult access to health care [3–5]. The diagnosis of BU is based on epidemiological and clinical criteria defined by the World Health Organization (WHO) [2,4]. The epidemiological WHO criteria are (i) residence or stay in a known BU endemic area and (ii) age between 0 and 15 years old since over 50% of all BU patients in Africa are children [6–8]. The clinical WHO criteria are (i) lesions on the upper or lower limbs since these represent about 85% of BU cases [4,8–11]; (ii) painless nodules, plaques or edema of the skin that, without treatment, evolve to a necrotic ulceration with [4,8] (iii) undermined and often hyperpigmented edges; (iv) lesions that are generally not accompanied by adenopathy, nor fever, and (v) that may become painful in case of superinfection [2,4].

Among the four tests recommended to confirm a BU diagnosis, two are most often used: direct smear examination (DSE) to detect acid-fast bacilli (AFB) and IS*2404* PCR, which is the most sensitive test to date yet with associated delays in the availability of results of more than 10 days [12–14]. Despite having the highest sensitivity of all laboratory tests, PCR does not detect all BU cases. Patients have been described that fulfill the epidemiological and clinical WHO criteria yet repeatedly test negative by PCR [7,15–17]. Given the limited sensitivity of PCR to confirm BU, and to avoid under-treatment of BU if relying on laboratory confirmation, the WHO recommends national BU programs to initiate treatment with BU-specific antibiotics even in the absence of confirmation guided by the WHO clinical and epidemiological criteria described above [4]. However, WHO does recommend to enforce the laboratory confirmation of BU, aiming for at least 70% of notified BU cases to be confirmed by a positive PCR result [12]. In the current context of a declining BU incidence observed in several endemic countries with good surveillance in place, and consequently a proportional increase of non-BU lesions being treated in BU facilities [18,19], we expect waning clinical expertise in the recognition of BU. This can result in diagnostic and therapeutic errors as has been observed for leprosy [20].

Smelling may be among the oldest diagnostic methods, as different pathologies, such as infectious and endogenous metabolic disorders, can affect human body odors [21]. A study in Cameroon found the characteristic smell of BU to be strongly associated with a confirmed BU diagnosis and described it as the smell of rotten fish, cassava or cheese, mixed with that of pyocyanic bacteria [22]. In our clinical experience, this characteristic smell draws our attention to a BU diagnostic, even in atypical presentations.

We recently reported on the accuracy of the clinical and microbiological diagnosis of BU and found that clinicians recognized BU with a sensitivity of 92% (95%CI 85%-96%) which was higher than the sensitivity of any of the laboratory tests. However, 14% (95%CI 7%-24%) of patients not suspected to have BU at diagnosis were classified as BU by a clinical expert panel [17]. We have therefore further investigated the WHO clinical and epidemiological criteria of the cohort of patients with lesions compatible with BU from our previous study and explored how these could be improved.

## Methods

### Ethical statements

The study was approved by the Provisional National Committee for Ethics in Health Research of Benin (registration n°: IRB 00006860), the Institutional Review Board of the ITM (code: 11 25 4 778) and the Committee for Medical Ethics of the Antwerp University Hospital (registration n°: B300201213080). The study also received an administrative authorization of the Benin Ministry of Health Ethics Board (N°IORG 0005695). All patients included in the study provided informed written consent. Parents or guardians provided consent on behalf of their children if participants were under the age of 18.

### Study type, population, location, and sampling

This is an analytical prospective study of 224 consecutive patients presenting with skin and soft tissue lesions that could be compatible with BU (nodules, plaques, edemas, ulcers or osteomyelitis, including those recognized as unlikely BU by experienced clinicians), living in a BU endemic region, who were recruited in the “Centre de Dépistage et de Traitement de l’Ulcère de Buruli” (CDTUB) of Allada and Lalo in southern Benin between March 2012 and March 2015. Traumatic lesions of less than two weeks duration and relapses of BU were excluded from the study. Depending on whether the clinical and epidemiological characteristics of BU were met, the lesions were diagnosed clinically as BU or non-BU by experienced clinicians who had been trained on the WHO BU diagnostic criteria. Two swabs were taken from ulcers, or two fine-needle aspirates from closed lesions for the microbiological confirmation by IS*2404* PCR and DSE after auramine staining in the Mycobacteriology Reference Laboratory in Cotonou (Benin), with quality control for molecular analyses performed by the Institute of Tropical Medicine in Antwerp (Belgium).

### Variables, data collection and statistical methods

The epidemiological, clinical and microbiological results of the patients were collected using standard WHO forms and entered in a Microsoft Access database by dedicated staff. We collected data related to the WHO diagnostic criteria (age, type and location of the lesion, pain, fever, adenopathy) and additional clinical and epidemiological information (size of lesion, WHO category, gender, and functional limitation). These results were documented for each patient by a team of two clinicians and three nurses, experienced in the diagnosis, treatment and management of BU. The presence of a characteristic smell was discussed systematically between clinicians at the time of sampling, before treatment initiation. The clinical team was not aware of laboratory information (not available yet at the time of clinical examination) but was unblinded to clinical and epidemiological information.

The statistical analysis was done using Epi Info 7.2.2.6 (Database and statistics software for public health professionals, Centers for Disease Control and Prevention (CDC), Atlanta, USA) and STATA/SE 11.0. As a reference standard for BU confirmation we used IS*2404* PCR. We performed univariate, bivariate and multivariate analysis using logistic regression with PCR result as dependent variable in order to establish a predictive model for BU. We also tested for interaction and confounding among variables associated with the PCR result using stratified analysis in bivariate analysis. All variables associated with a positive PCR result in the univariate analysis with a p-value <0.10 were considered in the multivariate model. We used a backward elimination procedure, probability for removal was set at <0.05. Odds ratios (OR) and their 95% confidence intervals (95%CI) were used as a measure of strength of the associations with a positive PCR result. We studied two predictive models of a positive PCR result and estimated the discriminative ability of both predictive models using the ROC analysis.

## Results

Among the 224 participants included in this study, 120 (53.6%) were male and 108 (48.2%) were ≤15 years old. Median age was 18 years (IQR: 9–42 years). A total of 201 (89.7%) patients had ulcerated lesions and 201 (89.7%) had lesions on their limbs. A clinical BU diagnosis was made in 134 (59.8%) patients. PCR was positive for 98 (43.7%) participants among whom 9 participants had been clinically diagnosed as non-BU (10.0% of patients diagnosed as clinically non-BU). DSE was positive for AFB for 37 participants (16.6%) (Table 1).

**Table 1:** Clinical characteristics and their association with positive PCR results.

Variables	Total (%) n = 224	PCR+ (%) n = 98	PCR- (%) n = 126	OR (95%CI)	p-value
**WHO clinical and epidemiological characteristics**					
Age ≤ 15 years	108 (48.2)	70 (71.4)	38 (30.2)	5.8 (3.2–10.3)	< 0.001
Located on limbs	201 (89.7)	87 (88.8)	114 (90.5)	0.8 (0.3–2.0)	0.678
Located on lower limbs	146 (65.2)	54 (55.1)	92 (73.0)	0.4 (0.3–0.8)	0.006
Located on upper limbs	55 (24.5)	33 (33.7)	22 (17.5)	2.4 (1.3–4.5)	0.006
Absence of pain	134 (59.8)	84 (85.7)	50 (39.7)	9.1 (4.7–17.8)	< 0.001
Absence of fever	212 (94.6)	96 (98.0)	116 (92.1)	4.1 (0.9–19.3)	0.071
Absence of satellite adenopathy	223 (99.5)	98 (100.0)	125 (99.2)	NA	NA
Ulcerated lesion	201 (89.7)	87 (88.8)	114 (90.5)	0.8 (0.3–2.0)	0.678
Necrotic base (n = 199)^a^	165 (82.9)	82 (95.3)	83 (73.4)	7.4 (2.5–22.0)	< 0.001
Undermined edge (n = 194)^a^	143 (73.7)	75 (89.3)	68 (61.8)	5.1 (2.3–11.3)	< 0.001
**Additional clinical and epidemiological characteristics**					
Characteristic smell (n = 190)^a^	107 (56.3)	74 (88.1)	33 (31.1)	16.4 (7.5–35.6)	< 0.001
WHO category of lesion (n = 223)^a^					
WHO Category 3 (diameter > 15cm)	81 (36.3)	27 (27.5)	54 (43.2)	1	-
WHO Category 2 (5 < diameter ≤ 15cm)	111 (49.8)	60 (61.2)	51 (40.8)	2.35 (1.30–4.26)	0.005
WHO Category 1 (diameter ≤ 5cm)	31 (13.9)	11 (11.2)	20 (16.0)	1.1 (0.46–2.62)	0.830
Male sex	120 (53.6)	51 (52.0)	69 (54.7)	0.9 (0.5–1.5)	0.685
Functional limitations	96 (42.9)	41 (41.8)	55 (43.6)	0.9 (0.5–1.6)	0.786
**Diagnostic test**					
Initial clinical BU diagnosis	134 (59.8)	89 (90.8)	45 (35.7)	17.8 (8.2–38.7)	< 0.001
Positive direct smear examination (n = 223)^a^	37 (16.6)	37 (38.1)	0 (0.0)	NA	NA

^a^The number of patients with available data varies because of missing data

On bivariate analysis we confirmed that most of the WHO clinical and epidemiological criteria of BU were indeed associated with a positive PCR result (Table 1). Age ≤ 15 years was significantly associated with a positive PCR result with an OR of 5.8 (95%CI: 3.2–10.3). Painless lesions were also significantly associated with a positive PCR result with an OR of 9.1 (95%CI: 4.7–17.8). For factors related to ulcerated lesions such as “necrotic base” and “undermined edge”, the associations were also strong and statistically significant with an OR of respectively 7.4 (95%CI: 2.5–22.0) and 5.1 (95%CI: 2.3–11.3). There was no statistically significant association with the localization of the lesions on the upper or lower limbs (OR: 0.8, 95%CI: 0.3–2.0) while a localization on the lower limbs was negatively associated with a PCR confirmed BU (OR: 0.4, 95%CI: 0.3–0.8) and a localization on the upper limbs was positively associated with a PCR confirmed BU (OR: 2.4, 95%IC: 1.3–4.5). There was no association with “absence of satellite adenopathy” and “absence of fever”.

Among the additional characteristics studied, the characteristic smell of ulcerated BU lesions was strongly associated with a positive PCR result with an OR of 16.4 (95%CI: 7.5–35.6), as was the initial clinical BU diagnosis made by the clinicians (OR: 17.8, 95%CI: 8.2–38.7), essentially a synthesis of the WHO criteria and clinical experience. A lesion size between 5–15 cm (WHO category 2) had a significant positive association with a positive PCR result with an OR of 2.35 (95%IC: 1.30–4.26). Gender and functional limitation were not associated with a positive PCR result (Table 1). There was no effect modification among the biologically plausible interactions we tested for (characteristic smell/necrotic base and characteristic smell/WHO category 3).

In a multivariate predictive logistic regression model exploring the associations between the WHO criteria and a positive PCR result, only “age ≤ 15years”, “absence of pain” and “necrotic base of ulcerative lesion” were retained with ORs of respectively 3.3 (95%IC: 1.6–6.7), 5.9 (95%IC: 2.7–12.8) and 3.7 (95%IC: 1.1–12.2). In a second multivariate predictive model, exploring all variables associated with a positive PCR result at a p <0.10 on univariate analysis, only the clinical criteria “characteristic smell”, “necrotic base” and “WHO category 2” were retained (Table 2).

**Table 2:** Predictive model of positive PCR result based on WHO criteria (model A) and predictive model of positive PCR result based on WHO criteria and additional characteristic (model B).

Variables	OR	95% C.I.	p-value
**Model A** (included variables: age, absence of pain, absence of fever, localization, necrotic base, undermined edge)			
**Absence of pain**	6.3	2.8–14.2	< 0.001
**Necrotic base**	3.9	1.2–12.8	0.026
**Age ≤ 15 years**	2.7	1.3–5.7	0.007
**Model B** (included variables: age, absence of pain, absence of fever, localization, necrotic base, undermined edge, characteristic smell, WHO category).			
**Characteristic smell**	15.7	6.6–37.4	< 0.001
**Necrotic base**	3.8	1.0–14.2	0.046
**WHO Category**			
WHO Category 3	1	-	-
WHO Category 2	2.5	1.1–5.7	0.032
WHO Category 1	0.4	0.1–1.4	0.153

**Fig 1:**
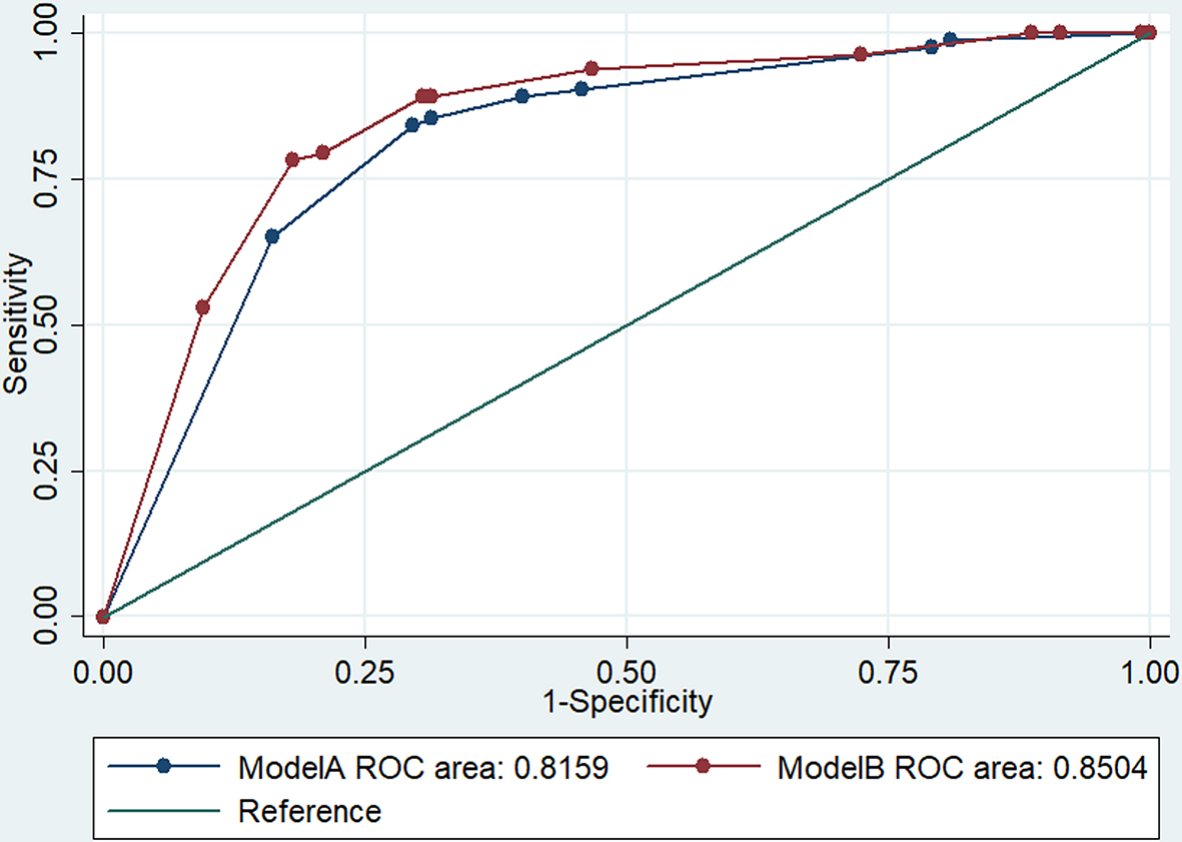
ROC analysis for both model A and model B.

To estimate the discriminative ability of both predictive models A and B, we estimated the areas under their ROC curves and found that model A (AUC: 0.82, 95%CI: 0.76–0.88) discriminated equally well as model B (AUC: 0.85, 95%CI: 0.79–0.91) between BU and non-BU patients (p = 0.1940) (Fig 1).

## Discussion

Our study results confirm the validity of the WHO epidemiological and clinical criteria to guide the diagnostic process for BU. However, we found that the localization of lesions on the lower limbs and WHO category 3 lesions are inversely associated with a positive PCR result. In addition, the characteristic smell of ulcerated lesions recognized by experienced clinicians was an even stronger predictor. We also showed that even experienced clinicians can miss the diagnosis of BU in PCR confirmed BU patients.

Most of the epidemiological and clinical criteria for a BU diagnosis defined by WHO [4] were associated with a positive PCR result with varying OR’s aligning with the clinical and epidemiological description of BU patients in literature [2,3,12,22,23]. The localization of lesions on the limbs was not associated with a positive PCR result and was therefore not predictive of BU. As also mentioned by the WHO [4], lesions on the lower limbs are more prone to being PCR negative than those on the upper limbs or other parts of the body and we indeed found them to be negatively associated with a PCR confirmation. Kibadi et al. [15] made a similar observation in a study including clinically suspected BU patients with large ulcers. Among their 25 PCR negative patients, 23 had lesions on the lower limbs. It is thus likely that these PCR negative patients did not have BU. Lesions on the lower limbs have a broader range of etiologies with proportionally less BU [4,24]. The clinical suspicion for BU can moreover be broadened since 10.0% of patients clinically diagnosed as non-BU in our study turned out to have BU by PCR, as we reported before [17].

We found the characteristic smell of ulcerated BU lesions to be strongly associated with a PCR confirmation, suggesting that it is specific for BU. This was also reported by Mueller et al. in a BU treatment centre in Cameroon [22]. In our clinical experience, this characteristic smell, distinct from the smell of “putrid” wounds, draws our attention to a BU diagnosis even in atypical presentations. Moreover, this characteristic odor can be sensed during surgical excisions of non-ulcerated BU lesions, particularly on edematous lesions. According to Mueller et al. the characteristic BU smell was described by clinicians as strong, like the smell of rotten fish, cassava or cheese, mixed with that of pyocyanic bacteria [22]. Ribera et al. described this smell as “unpleasant” and stated that it was recognized also by the BU patient's family and is a stigmatizing factor [25]. In a short survey we held among 15 health care workers from the CDTUBs of Allada and Lalo, they all recognized that ulcerative lesions of BU have a characteristic smell and that this smell differs from the smell of other chronic wounds. However, the description varied between disagreeable, nauseating, strongly penetrating and rotten. Specific smells have been described in other pathologies [26–28] and, if reproducible and validated by experienced clinicians, the BU specific volatiles could be an important tool to train health care workers, especially in the current context of a decreasing BU incidence [29]. Capturing such specific volatiles could allow the development of a Point-of-Care diagnostic test and thus improve BU diagnosis in the field [30,31].

In a preliminary study, we analyzed the organic volatiles in used gauzes of bandages of 13 presumptive BU and 17 non-BU patients using a hand-held chemical vapor sensing instrument which predicted the clinical BU diagnosis in 66.7% of samples [32]. For tuberculosis, the recognition of specific volatiles from sputa by trained Gambian pouched rats has been shown to be a helpful diagnostic test [26], although we are not aware of publications on clinicians distinguishing tuberculosis patients from those with other pulmonary diseases based on smell. In order to test whether smell could be used as a diagnostic tool, *M*. *ulcerans* volatiles should be characterized in vitro, in parallel with characterization of air sampled in operation theaters, with appropriate controls. This would allow the development of an «electronic-nose» that could be useful as a non-invasive diagnostic tool that can be easily used during active case-finding activities, as for other diseases [33–35]. In carcinoma diagnosis for example, an electronic-nose was used to differentiate head and neck carcinoma from lung carcinoma [36] and also to discriminate head and neck squamous carcinoma from colon and bladder carcinoma [37]. An electronic-nose allowed the detection of cannabis use on the human skin surface [38]. A non-invasive test based on smell will only be applicable to ulcerated lesions, although for the differential diagnosis of ulcerated forms which make up the majority of BU compatible lesions, a smell-based Point-of-Care test could contribute to the discrimination between BU and non-BU lesions. Early non-ulcerated lesions are expected to become more common with increasing BU control efforts and will not be testable by an electronic nose unless after validation on fine-needle aspirations.

Apart from the characteristic smell, lesions with diameter between 5–15 cm (WHO category 2) were more likely PCR positive while lesions with diameter > 15 cm were inversely associated with a positive PCR. This might result from the impact of BU control strategies focusing on early detection of BU lesions [39] by involving community volunteers in active case-finding [40,41], as BU lesions can be quite extensive when detected late. Another explanation could be that in large ulcers the bacterial load reduces due to the natural course of spontaneous healing of BU resulting in lower PCR confirmation rates.

Limitations of this study include the fact that our reference standard of BU diagnosis, PCR, is not perfect although it is currently the best available test [12,17,42,43]. The proportion of confirmed BU in our population of suspected BU patients may have been higher than what would be seen among patients suspected of having BU in routine clinical practice in an endemic area since the prevalence of BU is probably higher in the referral centers of our study. Moreover, the clinicians of this study are probably more experienced in recognizing BU clinically. This could lead to overestimating the discriminative value of the predictive model. Strictly speaking, our model is thus predictive of PCR positive BU rather than of a BU diagnosis. With regards to specificity, quality controls on the molecular laboratory performing the IS*2404* PCR consistently yielded excellent results. Our team of clinicians and nurses evaluating the patients was not blinded to clinical and epidemiological information when identifying the characteristic smell, possibly introducing a bias in their appreciation of the BU specific volatiles. However, blinding the clinicians to features of the lesion that in clinical practice are observed anyway, would underestimate the true accuracy of smell, resulting in test review bias [44]. The estimates of smell accuracy in such an experimental setting would not mimic its performance in a clinical setting. Also, the inter-observer variability in identifying the characteristic BU smell cannot be analyzed in this dataset.

### Conclusion

The current BU diagnostic criteria can benefit from revision by differentiating between lesions on the upper and lower limbs and by including lesion size and the characteristic smell recognized by experienced clinicians. Although the characteristic BU smell, strongly associated with a positive PCR result, will be difficult to describe in guidelines that need to be understandable to non-experienced clinicians. Further studies need to clarify if *M*. *ulcerans* indeed releases specific volatiles that can serve for the development of Point-of-Care diagnostic tests useful for non-invasive confirmation during active case-finding activities. Reproduced BU volatiles, if safe, may moreover serve for training purposes. Both could be important tools for health care workers, especially in the present context of decreasing BU incidence.

## Supporting information

S1 Text Supporting data. (XLSX)

S2 Text STROBE checklist. (DOC)
